# Closing the loop: short term impacts on physical activity of the completion of a loop trail in Sydney, Australia

**DOI:** 10.1186/s12966-019-0815-4

**Published:** 2019-07-15

**Authors:** Anne Grunseit, Melanie Crane, Paul Klarenaar, Jonathon Noyes, Dafna Merom

**Affiliations:** 1 The Australian Prevention Partnership Centre, Sydney School of Public Health, Level 6, Charles Perkins Centre, University of Sydney, Camperdown, NSW 2006 Australia; 2 Sydney School of Public Health, Level 6, Charles Perkins Centre, University of Sydney, Camperdown, NSW 2006 Australia; 3 Northern Sydney Local Health District Health Promotion, Brookvale Community Health Centre, Level 4, 612-624 Pittwater Rd, Brookvale, NSW 2100 Australia; 4 Physical Activity and Health, School of Science and Health, Western Sydney University, Sydney, Australia

**Keywords:** Natural experimental, Physical activity, Recreational trail, Built environment, Intercept survey, Time series

## Abstract

**Background:**

In Australia, an estimated 57% of the population do not meet physical activity recommendations for health. The built environment is important for active living, and recreational trails provide safe and pleasant settings for this purpose. However, evidence for positive impacts on physical activity from real world natural experiments is sparse. We describe the impact of transforming a recreational trail into a loop on usage by cyclists and pedestrians and users’ physical activity levels.

**Method:**

We conducted time series analyses of pre and post-completion (November 2013–July 2015) counts taken from infrared electronic counters of pedestrians and cyclists on two established sections of the trail adjusted for underlying trend, trend change, weather, holidays and trail closures. Chi-square analyses of pre and post-completion visual counts examined change in the distribution of pedestrian/cyclist, adult/child, and male/female users. Descriptive and bivariate analyses of post-completion intercept survey data of 249 trail users were conducted to examine user characteristics and impact on physical activity.

**Results:**

Pedestrian and cyclist counts on established trail sections increased by between 200 and 340% from pre to post-completion. Visual count data showed a significant 7% increase in children (vs adults) using the trail at one site pre to post (*p* = 0.008). Of previous users, 48% reported doing more physical activity at the trail and this was additional to (not replacing) physical activity done elsewhere. Those users not meeting physical activity recommendations were more likely to report increased total physical activity since the loop was created (55.5% vs 39.2%, *p* = 0.031). The connected loop nature of the trail and its length was perceived to encourage more and different forms of physical activity.

**Conclusion:**

Creating an accessible loop trail away from motorised traffic can lead to increased trail use and potentially total physical activity. The modification to the trail encouraged proportionate and real increases in usage among vulnerable populations such as children and perhaps greater total physical activity especially for people not meeting physical activity recommendations. The findings suggest that the benefits of environmental changes such as these can accrue to those most in need of support for being physically active.

**Electronic supplementary material:**

The online version of this article (10.1186/s12966-019-0815-4) contains supplementary material, which is available to authorized users.

## Highlights

- Modifying a recreational trail to 8.5km loop increased more than tripled usage
- Pedestrians and cyclists counts increased despite little promotion
- Proportional and real increases in child users were observed
- Physically inactive people in particular increased their total physical activity

## Background

Physical inactivity is linked with poor health outcomes, including chronic diseases such as diabetes, cancer, osteoarthritis and cardiovascular disease [1] and is the fourth leading cause of death globally [2]. In Australia, an estimated 57% of the adult population and 81% of children aged 5–17 years are not meeting physical activity (PA) recommendations to maintain health [3]. Successfully intervening to increase the proportion who are sufficiently active at the population level is likely to involve multiple PA domains (active commuting, recreational, occupational) and levels (individual, community, societal) for greater reach and sustainability [4]. While there have been many studies examining PA and health, only a small minority are intervention studies [5].

The built environment can have a strong influence on active living and positive health outcomes [6]. For example, residential proximity to recreation facilities, access to parks and trails (publicly accessible walking/cycling paths through green space) have been associated with higher PA and better health outcomes [7]; cross-sectional evidence shows greater amounts of PA among trail users compared with non-users [8]. However, different built environment features may be important for recreation as opposed to transport activity. One prospective Australian study examining neighbourhood factors and uptake of active travel showed that more walkable neighbourhoods and those with good connectivity were associated with uptake of recreational cycling but not for transport [9]. Yet, research conducted in the UK validating a scale on perceptions of the physical environment and their relationship to PA, found that cycling for transport was only associated with street connectivity, and environmental perceptions were unrelated to recreational cycling [10]. Walking for recreation on the other hand was associated with supportive infrastructure, and walking for transport was additionally associated with availability of local amenities and general environmental quality [10]. Broader context also is important when assessing the relationship between environment and PA. For example, despite high bike ownership in Australia, this has not translated to high usage [11] and the number of bike trips made is still extremely low when compared to other countries [12]. A general lack of separated (from motorised traffic) cycling infrastructure and related concern for safety have been cited in previous research as reasons for the low cycling participation rates in Sydney, Australia’s most populous city in particular [13, 14]. There is also a need to better understand how these environmental factors impact on vulnerable and underactive populations such as children and women.

Prospective studies on the impact of new infrastructure to support PA have varied in terms of outcomes. The iConnect group in the UK found increases in walking and cycling activity with the opening of a new multi-use trail [15] and two studies in the US have shown that improved neighbourhood connectivity through building a greenway or trail significantly increased total walking and/or cycling in intervention neighbourhoods compared with control neighbourhoods [16, 17]. The establishment of a separated cycleway in a densely urban area in Australia also led to increased bike counts in the area and in self-reported cycling frequency in a cohort of residents living close to the new infrastructure, however no significant changes in overall PA were observed [18]. Burbidge and Goulias (2009) similarly found almost no evidence of change in PA following installation of a multi-use trail. Those who did increase in overall PA episodes, did not specifically walk or cycle, indicating that their activity was unrelated to the trail infrastructure [19]. Other studies have shown an increase in active travel in response to new urban infrastructure, however no change in overall PA [20, 21]. This may imply that people were substituting activity at the new infrastructure for that undertaken elsewhere, but might also suggest that the time from construction to when the study was conducted and the type of infrastructure (whether single or multi-use path) and how it is used has a differential effect on behaviour [22]. From the above, it appears that impacts vary with the type of environmental change and the outcome of interest, along with the wider context in which the infrastructure is situated [23, 24].

Evaluations of infrastructure which support PA are important as they serve to build the business case for investment in such developments and provide information to assist in local government planning and health promotion policy [25]. However, the ability to causally attribute changes in PA to the built environment has been limited by study quality and reporting of methods [26, 27]. We would contend that prospective studies through natural experiments such as those described above provide good evidence for causality and have the added advantage of being contextualised in the real world, rather than researcher driven. Recently, some authors have argued that such studies, when systematically assessed, suffer biases from a multitude of sources thereby undermining their capacity to establish a causal link between exposure and outcome [28, 29]. Benton et al. (2016), for example, examined twelve natural experiments evaluating the impact of changes to the built environment on PA, finding all outcomes had a critical risk of bias; sources of bias were most often due to poor control of confounders and ill-matched control sites. A subsequent commentary of this analysis [30], commended the push for sound methodological practice, but noted that natural experimental studies often take place under challenging budget, time and practical constraints limiting the opportunity to deploy ideal methodological designs. Therefore, in order to better understand the influence of context, it is important that as many evaluations are conducted as opportunities become available, whilst maximising the design as far as is practicable to enhance the evidence base. This paper focuses on one such evaluation of the impact of the completion of a recreational trail located in a suburb of Sydney, Australia on PA and trail usage, including the use by different subpopulations.

## Methods

### Setting

**Fig. 1:**
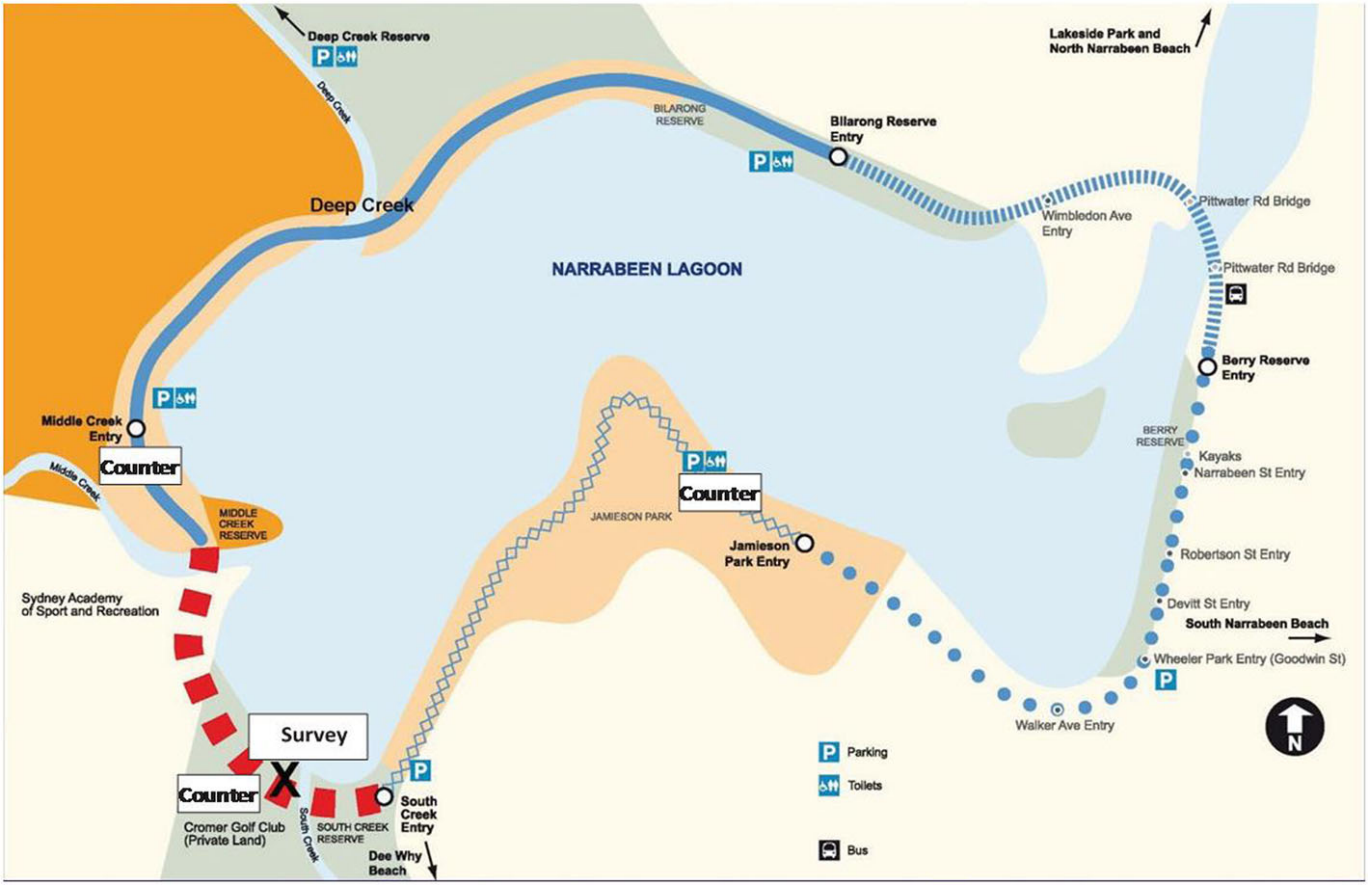
The Narrabeen Lagoon trail with the new section marked in red and intercept survey and counter locations

The Narrabeen Lagoon Trail is a multi-use recreational walking and cycling loop trail (a trail which connects back with itself when travelling continuously in one or the other direction) located in a densely populated area of Northern Sydney, Australia. It runs through bushland, parks and passes by amenities such as parking areas, other recreational activities (ie., watercraft hire) and cafes/restaurants. The trail has been undergoing development since 2010 with the final stage of the trail opening on 25th February 2015. Completion of the final section means that the 8.5 km trail fully circumnavigates Narrabeen Lagoon linking the suburbs of Narrabeen and Cromer from both directions, providing a trail for pedestrian and cyclist use that is entirely off-road (see Fig. 1). The local council implemented the project which involved building new bridges, 2 km of new boardwalk, reserve and car park upgrades, a boat ramp, toilet facility upgrades, park furniture, rest stops, vantage outlook points, heritage restoration, environmental protection and substantial planting of local vegetation at a cost of $AUD 11.4 million [31]. The trail was 60% funded by the then Warringah (now Northern Beaches) Council, and supported by State and Federal grants programs.

### Evaluation approach

The research was opportunistic at the instigation of the researchers following an approach made to Warringah Council. The evaluation comprised three components: time series analysis of electronic counter data November 2012 to July 2015 at two existing locations and the new section of trail; visual count of users at the existing and new sections of trail on two occasions pre and post-completion; and intercept surveys on two occasions post-completion. The study was approved by the University of Sydney Human Research Ethics Committee Approval #2014/751.

### Data collection and treatment

1. Ecounter data

The council had passive infrared pyroelectric counters (EcoCounter, model Eco-Multi) which have shown acceptable accuracy and reliability for pedestrian and bicycle counts at trails [32]. For pedestrian traffic, there is a small risk of undercounting where there is occlusion caused by pedestrians walking side by side and prams may be counted as bikes [32]. Bike counts have been found to be within 1.6% of manual counts [32]. Data were available for the period 3rd November 2012 to 14th July 2015. Counters were placed in three locations: Jamieson Park, Middle Creek Reserve (existing sections), and James Wheeler Parade (the new section) (Fig. 1). The positioning of the counters ensured capture all trail users (and only trail users) on that section of trail (i.e., “squeeze-points” where only people specifically using the trail are counted). Only the data from the two existing sections were used for this analysis as the focus was on the change in use from pre to post-completion. The two sites mark two different types of entry points to the trail which may affect usage: Middle Creek was close to bushland but entry was from a road with fast-moving (80 km/hr. speed limit) traffic. Jamieson Park was closer to a residential area with a connecting share path.

The counter data were in hourly format but were collapsed to weekly format for analysis. Validity checks were conducted according to recommendations [32] and data anomalies occurring on 23 days at Middle Creek were corrected according to established methods [33] (see Additional file 1 for further details). Data from the Australian Bureau of Meteorology for the closest measurement stations for daily total rainfall (Long Reef) and maximum temperature (Terry Hills) were merged with the trail data. Counts were also coded for public holidays, school holidays and trail closure (due to storm damage) as these were likely to affect patronage.

2) Visual counts

Visual counts took place at two points near the electronic counter locations on the existing trail sections pre- and post-completion, plus at a third point on the newly completed section (post-completion only). Counts were undertaken by trained observers on two separate (weekend) occasions for the same four consecutive hours (9 am-1 pm) prior to completion of the final trail section (11th of October and 7th December 2014), and three weekend occasions approximately six weeks (21st March 2015) and 12 and 13 months post baseline (October and November 2015 respectively). Observers followed detailed written protocols circulated upon recruitment, and received further in-person instruction immediately prior to the first observation period. Changes and additions to data collection rules were documented and communicated to all team members and quality was monitored during data collection by a senior investigator (AG). Two observers were stationed at the busier sites (Jamieson Park and the new section) counting a single direction each, and one observer was at the (less busy) Middle Creek site. An extra observer travelled between the sites to assist with validation and standardisation. Counts were broken down by gender of users, user type (pedestrian or cyclist), direction of travel (except on survey days), and adult or child using standardised data collection forms with definitions of the different user types and direction of travel. Counts were also conducted on the new section on the same day as the survey was conducted for the purpose of calculating survey response rates, but these counts did not distinguish direction of travel.

3) Intercept survey

Intercept surveys with adult users of the trail (age 18+ checked in introduction) were undertaken on two separate (weekend) occasions (21st March and 9th May 2015) between the hours of 9 am and 4 pm after completion of the final trail section. Attempts were made to ensure representativeness based on distribution of users in the visual count (see above, and Additional file 1: Table S3). The surveys took place on the newly completed section in a clearing just off the path within approximately 150 m of the electronic counters (Fig. 1), clearly marked for a voluntary survey taking place. The survey was interviewer-administered by one of four trained researchers following a standard protocol for interviewee approach, recording of refusals, consent process, assignment of unique IDs and survey administration. A senior researcher (AG) was present to ensure administration standardisation and to answer queries from participants and interviewers. After a short introduction, checking the age of potential participant and outlining the purpose and content of the survey, information was gathered on the demographic characteristics of users, frequency of use of the trail in general and the new section since it opened, details of their use of the trail on the day of survey including how they came to the trail and who accompanied them, their PA generally and at the trail, and opinions of trail maintenance and safety. Surveys took between 5 and 10 min to complete.

### Analysis

#### Ecounter

Interrupted time series (ITS) analyses were undertaken of the Ecounter data for each site (Middle Creek Reserve and Jamieson Park) for each user type (pedestrian/cyclist) by direction of travel (clockwise/anti-clockwise). Each model was first tested for stationarity using Dickey-Fuller [34] and Phillips-Perron unit root tests [35] and in all cases the null hypothesis of having a unit root was rejected. Autocorrelations and partial autocorrelations were examined using correlograms and the Q-statistic to include the appropriate autoregressive terms for each model. ARIMA (ARMAX [36]) models were then fitted adjusted for underlying trend, the change in trend post-loop completion, average daily rainfall, number of public holidays, and school holidays. Non-significant terms were progressively dropped from the model. The average maximum temperature, season, and closure of the trail (following storm damage in 2015) did not reach statistical significance in any of the analyses and were therefore not retained in the final models. The exception was the pedestrian anticlockwise count, where average maximum temperature was significant and retained in final model. Mean predicted values were derived from the time series models at the existing locations for the 19 weeks February 25th to July 14th (weeks 9 to 28) for each year 2013, 2014 and 2015 and, as recommended elsewhere [29], compared using linear regression with year as the independent variable to test for differences controlling for seasonality.

#### Visual counts

Visual count data were aggregated to form separate pre and post counts data for each user type (pedestrian/cyclist), direction of travel, gender, adult/child and location. Percentage increase from pre to post-completion were calculated for the mean counts for these two periods. Chi-square analyses were performed to assess change in distribution across user characteristics pre to post-completion in order to examine whether the change in infrastructure affected usage differentially by different subpopulations.

#### Intercept survey

Descriptive statistics were generated for the intercept survey samples combined for the two survey occasions. Two questions were analysed which measured PA generally and PA performed at the trail:

*In the past week, on how many days have you done a total of 30 min or more of physical activity which was enough to raise your breathing rate? This may include sport, exercise and brisk walking or cycling for recreation or to get to and from places but should not include housework or physical activity that is part of your job*. The number of days the participant reported was recorded. This question has been tested previously and found to be reliable and valid when compared to longer PA questionnaires [37].

The second (open-ended) question examined the impact of the modified trail on users’ PA at the trail, and included prompts as to whether the respondent did more (or less) PA at the trail than previously (if they were a user prior to the loop completion); and whether this was additional PA or replaced that done elsewhere:

*Since the new section opened, do you use the trail differently? That could be in terms of how often, how long, for what purpose, who you come with, where on the trail you go, or the type of activity you do when you are here?* Fixed response answers recorded “Using it the same way” if the respondent indicated no change, otherwise a free text box documented whether the person did more, less or different activity (e.g., used to run, now also cycles; now brings children to trail) at the trail. If more PA was indicated, notes were made whether it was additional or replaced PA done elsewhere. The question is similar to those used previously [38, 39]. Responses were post-coded for increase or decrease in activity and additional or replacing other activity by AG and cross-checked with the research assistant who administered the survey. Data on the change in PA at the trail since the loop was completed were missing for 15 people due to the research assistants failing to probe whether activity was additional or replaced other activity for these people. As the data were missing completely at random [40] this omission is unlikely to have affected the results except to reduce the power of the comparisons.

Analyses for all three data sources were conducted in Stata version 14.2 [36].

## Results

### Ecounter data

The results of the ITS analyses by location and user type for both existing sites are shown in Table 1. Results showed a similar pattern for the clockwise and anti-clockwise direction of travel so only the former are shown here and the latter are given in Additional file 1: Table S1 and any notable differences are described here.

**Table 1:** ITS results for clockwise direction for Middle Creek and Jamieson Park

Clockwise direction				
	Middle Creek		Jamieson Park	
Term	Bike	Pedestrian	Bike	Pedestrian
	Adj beta (95%CI)	Adj beta (95%CI)	Adj beta (95%CI)	Adj beta (95%CI)
Level change	1391 (1107, 1675)	1149 (9399, 1358)	1899 (1672, 2126)	812 (161, 1462)
Trend	0.4 (−1, 2)	0.4 (− 1, 2)	0.2 (− 1, 2)	3 (− 1, 7)
Trend change	− 50 (− 73, − 27)	−27 (− 43, − 10)	−62 (− 80, − 44)	−8 (− 42, 27)
Rainfall^1^	−4 (− 5, − 3)	−3 (− 4, − 2)	− 4 (− 5, − 2)	−2 (− 3, − 1)
Public holiday^2^	462 (343, 581)	245 (155, 336)	409 (298, 520)	165 (481, 282)
School holiday^3^	442 (291, 593)	470 (311, 628)	377 (241, 513)	356 (90, 623)

^1^ Weekly total rainfall in millimetres

^2^ Dichotomous coded 1 for when a public holiday fell in that week

^3^ Dichotomous coded 1 for when if school holidays fell in that week

Tables 1 and Additional file 1: Table S1 show there was no significant overall trend for either site or direction over the measured period, but a highly statistically significant level change from pre to post-loop completion. According to the ITS models, the average increase in the count of bike passes ranged from 1391 to 1899 (Table 1) per week in the post-loop completion period compared with pre-completion. For pedestrians, the average increase ranged from 756 (Additional file 1: Table S1) to 1149 (Table 1) per week. Importantly, there was also a significant change in trend pre- to post-opening for some (although not all) counts indicating a small but significant relative decreasing trend in counts after opening of the loop. Rainfall was a significant inverse predictor for bike and pedestrian counts, and public holidays and school holidays (except pedestrians at Middle Creek anticlockwise direction) were significant positive predictors in all models.

**Fig. 2:**
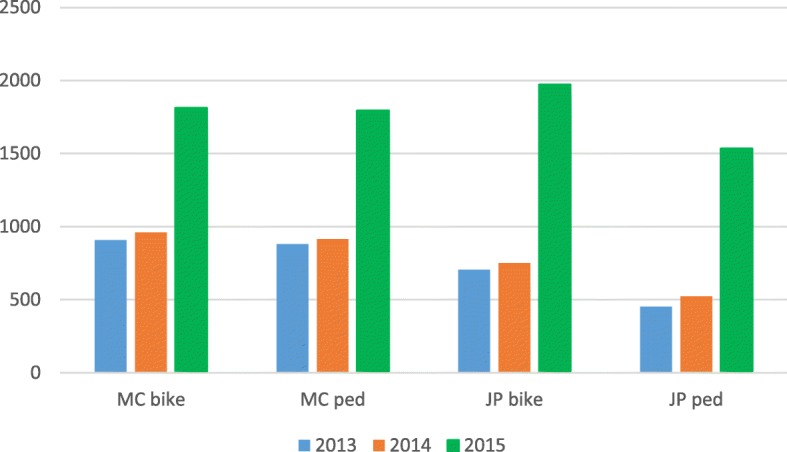
Adjusted counts of passes by travel mode (bike or pedestrian) clockwise direction of travel for Middle Creek (MC) and Jamieson Park (JP) electronic counter for Weeks 9 to 28 of calendar year 2013, 2014, 2015

Figure 2 (and Additional file 1: Figure S1), show the mean number of adjusted predicted bike and pedestrian passes in the same period (weeks 9 to 28) pre- (2013 and 2014 and post- (2015) loop completion. All mean counts were significantly higher in 2015 compared with 2013 and 2014 irrespective of direction of travel or user type (all *p* < 0.001). For example, the adjusted average number of passes by bikes approximately doubled at Middle Creek in 2015 compared with the same period in the preceding two years, and were 247 to 280% higher at Jamieson Park. Average adjusted pedestrian counts also approximately doubled at Middle Creek, but more than tripled (312 and 340%, anticlockwise and clockwise respectively) at Jamieson Park in 2015 compared with 2013.

### Visual count

**Fig. 3:**
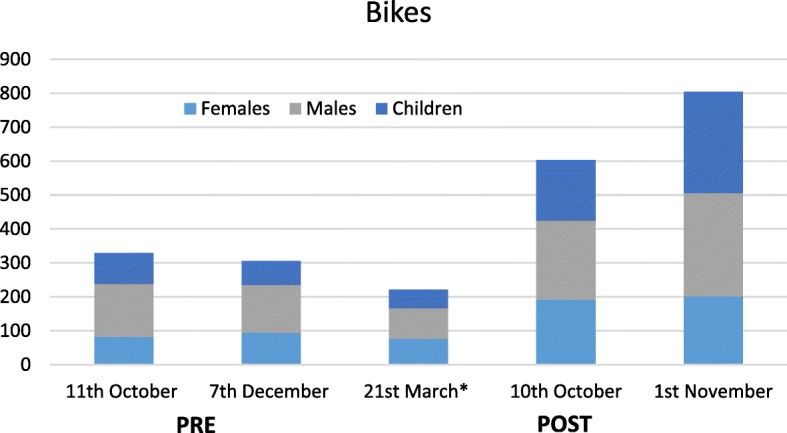
Distribution of bike users (female adults, male adults, children) aggregated across count sites by date of observation

**Fig. 4:**
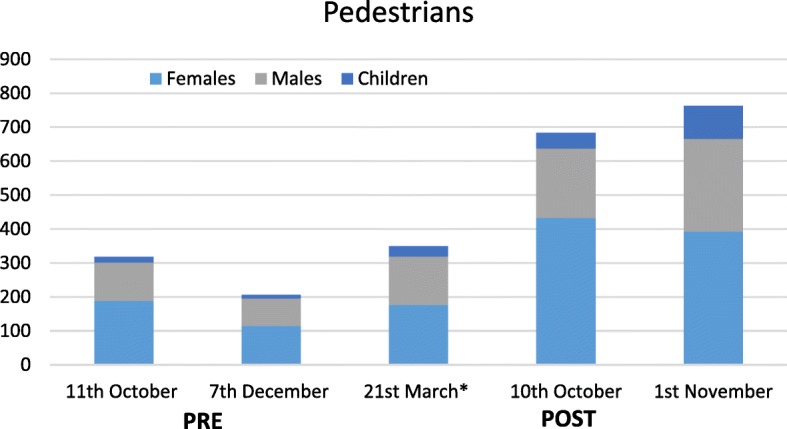
Distribution of pedestrian users (female adults, male adults, children) aggregated across count site by date of observation

Figures 3 and 4 show the distribution of user subpopulations on the five visual count occasions, collapsed over count site and direction of travel (Additional file 1: Table S2 in provides further detail broken down by site). The visual count data supported the increases in overall counts of users observed in the Ecounter data. In terms of the distribution of different subpopulations, there was a statistically significant change in distribution of male and female adult and children pedestrians and cyclists pre- to post-completion; the proportion of child cyclists increased from 5.3 to 9.6%, whereas adult males and females dropped by 2% each (*p* = 0.008). Among pedestrians, the proportion of children increased from 25.5 to 32.7% and the proportion of female pedestrians remained steady, but the proportion of male pedestrians dropped from 46.6 to 38.5% (*p* < 0.001) pre to post-completion.

### Intercept survey

A total of 114 and 135 trail users respectively agreed to be surveyed on the 21st of March and 9th of May 2015 respectively (both dates post-completion). The response rate was 61.9% of those approached over both days (Additional file 1: Table S3). Pedestrians were slightly over-represented in our sample (69.1%) compared with those passing the survey point on those days (59.6%), but gender distribution in the survey was close to representative (54.2% vs 51.7% female respectively).

Table 2 shows the survey sample distributions for demographic and other characteristics. Over half of the survey sample lived in the suburbs neighbouring the trail, while a third were from neighbouring council areas up to approximately 22 km away. Of all trail users, by far the majority of survey respondents travelled to the trail by car (66%), with 72% of pedestrian trail users (vs 52% of cyclist users) using this mode. A quarter of the pedestrians walked to get to the trail, and 45.5% of cyclists had cycled to the trail, meaning almost one-third of all users had actively travelled to the trail. However, of those who lived in suburbs neighbouring the trail (Elanora Heights/Narrabeen/North Narrabeen, Cromer/Dee Why, Collaroy) over half (51.6%) had travelled to the trail by either cycling or walking (Table 2).

**Table 2:** Intercept survey sample demographic characteristics and travel to trail by user type

	Pedestrian (*n* = 172)		Cyclist (*n* = 77)		All (*n* = 249)	
Gender	%	*frequency*	%	*frequency*	%	*frequency*
Male	39.0	67	61.0	*47*	45.6	*114*
Female	61.1	105	39.0	*30*	54.4	*135*
Age						
18–24	2.9	5	1.3	*1*	2.4	*7*
25–34	10.5	18	18.2	*14*	12.8	*32*
35–44	22.7	39	33.8	*26*	26.0	*65*
45–54	26.2	45	28.6	*22*	26.8	*67*
55–64	21.5	37	10.4	*8*	18.0	*45*
65+	16.3	28	7.8	*6*	13.6	*34*
Group size (adults + children)						
1 (adult only)	25.0	43	24.7	19	24.9	62
2	50.6	87	40.3	31	47.6	119
3	12.2	21	19.5	15	14.4	36
4	10.5	18	11.7	9	10.8	27
5	1.7	3	3.9	3	2.4	6
Residence						
Elanora Heights/Narrabeen, Collaroy, Cromer/Dee Why	54.4	93	38.2	29	49.2	122
Other SHOROC*	35.1	60	51.3	39	40.3	100
Not SHOROC*	10.5	18	10.5	8	10.5	26
How travelled to trail						
Car	71.5	123	52.0	40	65.6	163
Walking only	25.6	44	1.3	1	18	45
Cycling only	0.6	1	45.5	35	14.4	36
Public transport	1.2	2	0	0	0.8	2
Other	1.2	2	0	1	1.2	3
Time taken to travel to trail						
0–5 min	40.4	69	32.9	25	38.1	94
6–10 min	25.7	44	19.7	15	23.9	59
11–20 min	20.5	35	26.3	20	22.3	55
21–30 min	6.4	11	11.8	9	8.1	20
31+ mins	7.0	12	9.2	7	7.7	19

*Shore Regional Organisation of Councils including Pittwater, Warringah, Manly and Mosman Councils

On the days of the survey, two-thirds of the combined sample reported that they were travelling the full trail loop (approximately 8.5 km), more commonly among cyclist (80.5%) than pedestrian (59%) respondents. Among the 192 respondents who had used the trail previously, 48% said they were doing more PA at the trail since the new section opened and this was additional to their other PA. Only three people said they were doing less PA since the new section opened - two because they were now doing the full loop rather than a 10 km “out-and-back” route they used to take; the other said he/she used to cycle more but the trail was now too busy to do this. The remaining 50% reported that on balance, they were doing the same total amount of PA post- as pre-completion of the loop.

In terms of total PA, 58.5% of the sample were not reaching the recommended levels (i.e., 30 min of moderate-vigorous activity on fewer than 5 days). However, those who did not meet current guidelines were more likely than those meeting the guidelines to report doing more PA in total since the opening of the completed trail (55.5% vs 39.2% respectively, *p* = 0.031). When stratified by survey occasion, the proportion of insufficiently and sufficiently active respondents reporting doing more PA since the new section was completed were not significantly different at the first survey occasion 3.5 weeks after opening (60% vs 56.3% respectively, *p* = 0.742, *n* = 77). However, there was a significant difference at the second survey taken 10.5 weeks after opening (51.8% vs 27.7% respectively, *p* = 0.013, *n* = 103). Together, these findings suggest that as time passed and the opportunity to make use of the upgraded trail increased and the novelty effect diminished, the greater impact on total PA for the insufficiently active compared with those meeting PA guidelines became more pronounced.

## Discussion

Our evaluation provides some answers to key evaluation questions when assessing built infrastructure and health effects: “if you build it, will they come?”, and further if they come, who will come and will there be any PA benefit? That is, we sought to determine if is there an appreciable rise in PA in an environment which has been enhanced to better support it, is that increase among those who most need it, and does total PA increase among infrastructure users or does it merely replace PA which would have been done elsewhere? This natural experiment was a low cost, opportunistic study designed to describe these effects in an environmental modification where no other researcher-driven promotional activities took place. Increased trail activity reported post-intervention by cyclist and pedestrian users was corroborated by two objective methods taken pre- and post-intervention, namely electronic counters and observation. Further, completion of the loop trail appeared to have a measurable impact on PA of vulnerable groups, namely adults not currently meeting PA guidelines and children, and included supplementary rather than substitutive activity. Our results demonstrate the value of improving infrastructure for PA, especially where it is close to residential areas and provides a safe and pleasant environment for pedestrian and cyclists alike. Additionally, this is the first evaluation of a recreational walking and cycling trail with a loop design that we are aware of. Further detail of the implications of the findings are given below.

Two sources of objectively measured count data convincingly demonstrated large and sustained increases in trail usage by cyclists and pedestrians following the opening of the complete loop extension. Ecounter data recorded 200–300% increases in cyclist and pedestrian traffic, a magnitude of change which is unlikely to be attributable to external influences despite the absence of a control comparison site [30]. The observational count supported the Ecounter data. The average hourly weekend for pedestrian use (both directions) at the Jamieson Park evaluation site, before opening was approximately 56 pedestrians per hour, which gradually increased to 107 per hour eight months post-opening of the full trail loop. Although there was some evidence of a decreasing trend in usage post-completion once the change in mean number of users was taken into account, the post-completion data collection period also coincided with the commencement of Autumn and Winter when PA tends to decrease [41]. Further, visual count data collected approximately eight months after opening suggests the higher counts observed for bike and pedestrian traffic were largely maintained after the Ecounter data collection ceased.

In terms of change across different subpopulations, the increase in cyclists was lower than that observed for pedestrians. For both user types, however, proportionately more children had used the trail, especially in the existing Middle Creek area. The latter finding is noteworthy, as the Middle Creek entry to the trail is located away from residential areas, from a road with fast moving motorised traffic and previously had no particular features (toilets, barbecue facilities) to mark it as a destination. Once the new section was completed, there was comparatively easy and short access to Middle Creek from the residential area and connecting share path at the South Creek entry (≈1.2 km post-completion vs ≈ 7.3 km pre-completion), making it attractive across a range of physical capacities. The Middle Creek area was also upgraded with toilets and picnic areas, enhancing the location as a destination. Whilst this explanation could not be validated, this is a feature known to increase active travel [24, 42].

The intercept survey suggested a substantial increase in energy expenditure in adults as a result of the completion of the loop, as almost half reported doing additional (rather than substitute) activity at the trail since the loop was completed, thereby adding to their total PA. Further, the majority of the survey respondents reported covering the whole trail on the day of the survey; it may be those who might have otherwise travelled less than 8.5 km in an out-and-back session are drawn into travelling further because having a closed loop provides a natural and convenient “destination” [42]. Walking and cycling 8.5kms at a moderate-intensity (4.8kms/hour walk or bicycle 17kms/hour) equates to approximately 350 MET-minutes achieved by 1.5 h of walking or 30 min cycling [43]. This “dose” of PA is halfway to meeting the weekly minimum recommendation and therefore the Narrabeen trail can contribute substantially to users meeting Australian PA guidelines [44].

Importantly for public health, this natural experiment has demonstrated the benefit of upgrading infrastructure to important target populations, namely adults not meeting PA guidelines and children. Early studies on environmental interventions debated the “selection of users by activity level” whereby already active people would be more likely to select themselves into neighbourhoods conducive to PA [45]. Here we observed that trail use supported increases in PA for those most in need. First, the proportion of survey respondents who were ‘insufficiently active’ (58.5%) was somewhat higher than the state adult population estimate (42.1%) [46], suggesting the trail is attractive to this subpopulation. Second, the more time since the trail was completed, the wider the differences were in terms of doing more PA since the loop was completed between those who met PA recommendations and those who did not, in favour of the latter. These findings are consistent with previous research. For example, an evaluation of newly constructed trails of 12 miles in the USA indicated that the majority (77.5%) of trail users were habitually active exercisers prior to trail opening. However, nearly all (98%) of the “new” exercisers reported that their exercise amount had increased as opposed to only 50% of the habitually active [47]. In the UK, 30% of the users of the National Cycle Network thought that the trail made no difference to their activity levels, whereas 42% stated that the trail helped them to increase their activity “a lot” and 28% by “a small amount” [39]. Brownson et al. noted that in their evaluation of promotion of existing walking trails in Missouri Bootheel region 32% of trail users reported to have increased their PA since they started to use the trail, although the authors could not demonstrate an increase in 7-day walking rates compared with comparison sites [48].

However, perceptions of an increase in activity following an infrastructure change can be subject to “desirability bias”; using self-report PA questions not linked to trail use may overcome this bias, at least in part, and demonstrate evidence for impact. For example, the UK iConnect study used a longitudinal self-reported PA questionnaire, and found 19% of those not active and 35% under active (< 150 min walking/cycling in past week) at baseline reported use of the trail for recreation or transport [49]. Importantly, those living closer to the trail showed a net gain attributable to using the trail at two-year (but not one-year) follow-up demonstrating a likely causal relationship [15]. These and our own findings underscore the importance of having easily accessible infrastructure to support activity, especially for those who are less active, or groups who would be less likely to use areas open to motorised traffic such as children, women and older people [38, 50, 51]. Moreover, review evidence suggests that speed, movement, and momentum are important urban landform qualities for cycling, such as that provided by the loop design evaluated here [23].

Awareness has been raised as crucial antecedent to trail use in other studies, although not a guarantee of large impact [48]. In a new ‘Rail to Trail’ trail in Western Sydney, awareness of the trail among pedestrians living within 1.5kms to trail was 29% compared with 50% among cyclists living at the same distance [52]. In central North Carolina only 1% used or heard about the trail before end of construction or its official opening whereas two years later, 23.9% had both heard of the trail and used it at least once [53]. Our survey indicated that “word of mouth” as a source of awareness about the Narrabeen trail increased from 18.6% in the first survey to 25.2% in the second survey, whereas newspapers as a source dropped from 34 to 25% (Additional file 1: Table S4). This may suggest that awareness and therefore usage is likely to increase over time as community members’ social networks continue to disseminate knowledge about the trail beyond isolated media articles.

### Strengths and limitations

Several methodological merits of this study are worth mentioning. First, the triangulation of several methods in order to evaluate the impact of the intervention, a strategy recommended in the late 1990s [54] and more recently [30] for environmental interventions but rarely applied [26]. Second, we used two additional observational counts up to eight months after completion to assess the sustainability of the immediate effect established by the Ecounter data which ceased after four and a half months following completion [26]. Finally, the evaluation was a partnership between the local council, the Local Health District health promotion unit, and a university, which allowed sharing of knowledge, methods and insights and fostered research translation and the use of scientific evidence in local planning [30, 31].

However, there were a number of limitations to this study. The first was the inability to estimate trail awareness, usage and PA at the population level. The most common design to measure population level impact of a PA natural experiment evaluation is a pre-post survey estimating awareness, usage and changes in PA for residents with access to the trail [49, 52, 53]. However, these surveys are costly and were not an option for this opportunistic study. Second, we did not use a comparison location to account for natural trends over time [28]. However, given that we had over a year’s pre-completion Ecounter data, and up to eight months post-completion observational data, the observed increases in usage indicate a somewhat reliable temporal trend over a relatively long period.

With the objective data, Ecounters can only record “passes” and therefore the actual number of people using the trail is unknown. However, analysing passes separately by each direction can provide a proxy estimate of unique users. Change in PA was estimated retrospectively from qualitative responses to a question on change in PA with use of the trail which is yet to be validated, although has been used in previous natural experiment studies [38, 39, 53]. Variation in responses to this question (some people reported increasing their PA, others did not) demonstrates it likely reflects their actual behaviour rather than socially desirable responses. The intercept survey may have been biased towards capturing less active users as it was easier to recruit walkers rather than runners. However, we observed during the visual count and survey occasions far more walkers using the trail than runners and therefore it is likely that the survey does somewhat reflect the significant number of under-active people using the trail. We note that these limitations rank among those identified by Benton et al. (2016) and others [28, 29] as leading to serious bias and that this limits the confidence with which attributional conclusions may be made. However, the triangulation of data sources, the reliability of the objective methods used and the consistency of the pattern of results between both methods means this study provides useful evidence under the project for natural experiments as outlined by Humphreys et al. (2017) [30].

## Conclusion

Our evaluation of the impact of completion of an 8.5 km trail in Sydney, Australia showed a substantial increase in usage by pedestrian and cyclists users. The analysis suggests that those who are insufficiently active use the trail, which forms an important component of their total PA. The attraction of the trail has not diminished over time, and the increase in the proportion of children using the trail bodes well for inculcating a habit of being active in the local environment. As such, the study contributes to a broader understanding of the interaction between environment and PA and supports the case for providing pleasant and connected infrastructure in suburban locations.

## Additional file

Additional file 1: **Table S1.** Interrupted time series results for anticlockwise direction for Middle Creek and Jamieson Park. **Figure S1.** Adjusted counts of passes by travel mode (bike or pedestrian) anticlockwise direction of travel for Middle Creek (MC) and Jamieson Park (JP) electronic counter for Week 9 to 28 2013, 2014, 2015.**Table S2.** Visual count aggregated data for Middle Creek and Jamieson Park. **Table S3.** Response rate and representativeness of subpopulations for intercept surveys. **Table S4.** How respondent found out about new section of trail. (DOCX 26 kb)

## Data Availability

The Intercept Survey dataset used and/or analysed during the current study are available from the corresponding author on reasonable request. The visual count data are provided in Additional file 1. The Ecounter data that support the findings of this study are available from Northern Beaches Council but restrictions apply to the availability of these data, which were used under license for the current study, and so are not publicly available. Data are however available from the authors upon reasonable request and with permission of Northern Beaches Council.

## Abbreviations

ARIMA: Auto Regressive Integrated Moving Average
JP: Jamieson Park
MC: Middle Creek
MET: Metabolic Equivalent of Task
PA: physical activity
SHOROC: *Shore Regional Organisation of Councils including Pittwater, Warringah, Manly and Mosman Councils
