# Supplementary material for: Closing the loop: short term impacts on physical activity of the completion of a loop trail in Sydney, Australia
Source: Int J Behav Nutr Phys Act. 2019 Jul 15;16:57. doi: 10.1186/s12966-019-0815-4 (PMC6631862; doi:10.1186/s12966-019-0815-4)
Supplement: Supplementary file 1 — Table S1. Interrupted time series results for anticlockwise direction for Middle Creek and Jamieson Park. Figure S1. Adjusted counts of passes by travel mode (bike or pedestrian) anticlockwise direction of travel for Middle Creek (MC) and Jamieson Park (JP) electronic counter for Week 9 to 28 2013, 2014, 2015.Table S2. Visual count aggregated data for Middle Creek and Jamieson Park. Table S3. Response rate and representativeness of subpopulations for intercept surveys. Table S4. How respondent found out about new section of trail. (DOCX 26 kb) [file 12966_2019_815_MOESM1_ESM.docx]

**Additional File 1**

**Data anomaly correction**

There were a number of anomalies for the Ecounter data where the count for one direction (Middle Creek to Bilarong) would report as zero and count added to the opposite direction for both pedestrians and bikes. In order to correct for this, on days when this would occur the average ratio of one direction over the other for the entire period for that hour was applied to the total of both directions to apportion the counts to each direction. For example, if the ratio was 47% in direction A, and 53% direction B and the total count for that hour was 100, 47 would be credited to direction A and 53 to direction B. The ratio between the two directions did not vary between pre- and post-completion, day of the week, month, or year, but it did by hour, hence we averaged over the whole period by hour to obtain the appropriate ratios. There were also five days (29^th^ October 2014 to 2^nd^ November 2014) where the data for both directions were zero (total counter failure). These were imputed by taking the averages of the daily counts for the two preceding and succeeding corresponding days of the week as recommended by the Transportation Research Board [1].

Table S1: Interrupted time series results for anticlockwise direction for Middle Creek and Jamieson Park

| **Anticlockwise** | | | | |
| --- | --- | --- | --- | --- |
|  | **Middle Creek** | | **Jamieson Park** | |
| **Term** | **Bike** | **Pedestrian*** | **Bike** | **Pedestrian** |
|  | **Adj beta (95%CI)** | **Adj beta (95%CI)** | **Adj beta (95%CI)** | **Adj beta (95%CI)** |
| **Level change** | 1540 (1226, 1853) | 756 (542, 970) | 1828 (1590, 2066) | 788 (159, 1417) |
| **Trend** | 0.67 (-1, 2) | 0.44 (-1, 2) | 0.09 (-2, 2) | 3 (-1, 7) |
| **Trend change** | -64 (-92, -37) | -0.53 (-18, 17) | -65 (-85, -45) | -8 (-43, 27) |
| **Rainfall^1^** | -5 (-6, -3) | -2 (-3, -1) | -4 (-5, -3) | -2 (-3, -1) |
| **Public holiday^2^** | 487 (354, 620) | 292 (220, 364) | 431 (314, 548) | 177 (67, 287) |
| **School holiday^3^** | 470 (311, 628) | - | 348 (223, 472) | 383 (102, 664) |

^1^ Weekly total rainfall in millimetres

^2^ Dichotomous coded 1 for when a public holiday fell in that week

^3^ Dichotomous coded 1 for when if school holidays fell in that week

* School holidays was not significant so excluded from final model; final model also included mean maximum daily temperature beta=17 (95%CI: 3, 31)

Figure S1: Adjusted counts of passes by travel mode (bike or pedestrian) anticlockwise direction of travel for Middle Creek (MC) and Jamieson Park (JP) electronic counter for Week 9 to 28 2013, 2014, 2015.

Table S2: Visual count aggregated data for Middle Creek and Jamieson Park

|  | Subpopulation | **11th October 2014** | **7th December 2014** | **21st March* 2015** | **10th October 2015** | **1st November 2015** |
| --- | --- | --- | --- | --- | --- | --- |
| **Jamieson Park** | | | | | | |
|  | Pedestrian |  |  |  |  |  |
|  | **All** | **222** | **153** | **166** | **370** | **427** |
|  | Adult male | 78 | 59 | 62 | 113 | 155 |
|  | Adult female | 131 | 86 | 86 | 231 | 218 |
|  | Children | 13 | 8 | 18 | 26 | 54 |
|  | Cyclist |  |  |  |  |  |
|  | **All** | **221** | **195** | **112** | **309** | **407** |
|  | Adult male | 98 | 86 | 41 | 119 | 146 |
|  | Adult female | 52 | 63 | 41 | 96 | 107 |
|  | Children | 71 | 46 | 30 | 94 | 154 |
| **Middle Creek** | | | | | | |
|  | Pedestrian |  |  |  |  |  |
|  | **All** | **96** | **53** | **183** | **313** | **336** |
|  | Adult male | 35 | 21 | 80 | 91 | 118 |
|  | Adult female | 57 | 29 | 91 | 202 | 175 |
|  | Children | 4 | 3 | 12 | 20 | 43 |
|  | Cyclist |  |  |  |  |  |
|  | **All** | **108** | **111** | **109** | **294** | **397** |
|  | Adult male | 58 | 54 | 48 | 113 | 159 |
|  | Adult female | 30 | 32 | 36 | 96 | 94 |
|  | Children | 20 | 25 | 25 | 85 | 144 |

Table S3: Response rate and representativeness of subpopulations for intercept surveys

| **Subpopulation** | **Count** | **Approached** | **Refused** | **Surveyed** | **RR*** | **% of users** | **% of survey respondents** |
| --- | --- | --- | --- | --- | --- | --- | --- |
| **Pedestrians** | **1172** | **248** | **76** | **172** | **69.4%** | **59.6%** | **69.1%** |
| male | 478 | 91 | 24 | 67 | 73.6% | 24.3% | 26.9% |
| female | 694 | 157 | 52 | 105 | 66.9% | 35.3% | 42.2% |
| **Cyclists** | **796** | **154** | **77** | **77** | **50.0%** | **40.4%** | **30.9%** |
| male | 473 | 86 | 39 | 47 | 54.7% | 24.0% | 18.9% |
| female | 323 | 68 | 38 | 30 | 44.1% | 16.4% | 12.0% |
| All | 1968 | 402 | 153 | 249 | 61.9% | - | - |

*Response rate of those who were approached. Survey rate of adults passing survey point 12.7%.

Table S4: How respondent found out about new section of trail

| **How found out about new section** | **Survey 1 n %** | **Survey 2 n %** | **Total** |
| --- | --- | --- | --- |
| By using trail | 8 | 7 | 15 |
|  | 7.1 | 5.2 | 6.1 |
| Word of mouth | 21 | 34 | 55 |
|  | 18.6 | 25.2 | 22.2 |
| Noticed before or when using before | 42 | 50 | 92 |
|  | 37.2 | 37.0 | 37.1 |
| Newspaper | 38 | 34 | 72 |
|  | 33.6 | 25.2 | 29.0 |
| Council website | 0 | 3 | 3 |
|  | 0.0 | 2.2 | 1.2 |
| Others with me | 2 | 0 | 2 |
|  | 1.8 | 0.0 | 0.8 |
| Other | 2 | 7 | 9 |
|  | 1.8 | 5.2 | 3.6 |
| Total | 113 | 135 | 248 |

**REFERENCES**

1. Ryus P, Ferguson E, Laustsen KM, Schneider RJ, Proulx FR, Hull T, et al: *Guidebook on pedestrian and bicycle volume data collection.* 2014.
